# Under the Skin: A Case Series of Clostridium sordellii Necrotizing Soft Tissue Infections in Patients Who Inject Drugs

**DOI:** 10.7759/cureus.43870

**Published:** 2023-08-21

**Authors:** Victoria R Milano, M. Gabriela Cabanilla

**Affiliations:** 1 Pharmacy, University of New Mexico Health Sciences Center, Albuquerque, USA; 2 Internal Medicine Division of Infectious Diseases, University of New Mexico Health Sciences Center, Albuquerque, USA

**Keywords:** necrotizing infection, septic shock, clostridium sordellii, soft tissue infections, vulnerable populations

## Abstract

*Clostridium sordellii *has been well-associated as a cause of obstetric infections but is a less commonly recognized organism of necrotizing skin and soft tissue infections (NSTI). *C. sordelli* infections are rare with only 14 cases reported in the literature to date. These infections are often associated with profound septic shock and the mortality rate remains high. We report four patients who presented with *C. sordellii *NSTI over a period of two years at an academic medical center. Notably, injection drug use was the main risk factor for infection in these patients. Appropriate management of *C. sordellii* NSTI necessitates a combination of antibiotics and emergent surgical intervention. However, despite these efforts, the mortality rate remains high, with three of the four patients dying. Clinicians should consider NSTI in the differential diagnosis when evaluating skin and soft tissue infections in people who inject drugs. Furthermore, it is imperative to educate patients who engage in injection drug use on the potential risks associated with NSTI and inform them of warning signs that warrant immediate medical attention. The devastating consequences of *C. sordellii*-associated NSTI in this vulnerable population can be mitigated by enhancing awareness and facilitating early intervention.

## Introduction

*Clostridium sordellii* is a toxin-producing anerobic Gram-positive rod. Clostridia spores are more resistant to heat than other bacteria, making them difficult to kill by the brief heating injection drug users employ [1]. When the spores are introduced via subcutaneous or intramuscular injection, the focus of insoluble material, hemorrhage, and devitalized tissue at the injection site provides the anerobic environment that promotes clostridial growth [2]. Although *C. sordellii* is a known cause of myonecrosis and toxic shock syndrome in obstetric populations, there are limited data and case reports describing clostridial necrotizing infections attributed to injection drug use [3-4]. Here, we report four cases of *C. sordellii* necrotizing soft tissue infection treated at an academic medical center in Albuquerque, New Mexico, over a two-year period. Injection drug use was a risk factor for infection in all patients. The suspected source of infection was an abscess or soiled skin at the injection site, contaminated injection devices, or contaminated illicit substances. Overall, patient management consisted of surgical debridement and adjunctive antibiotic therapy but still resulted in a high mortality rate, with three of the four patients dying shortly after presentation.

## Case presentation

Case 1 

**Figure 1:**
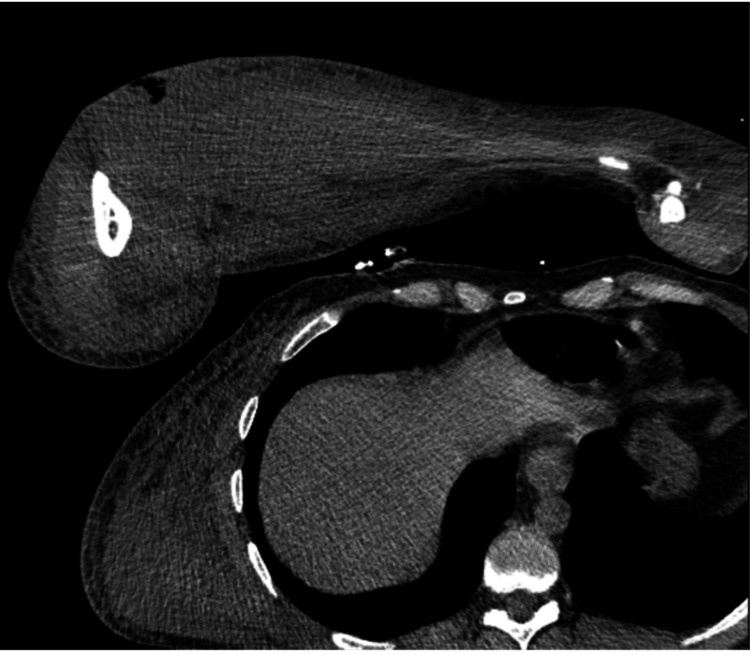
CT of the right upper extremity demonstrating subcutaneous and intramuscular edema of the right forearm with a small focus of gas within the proximal radial aspect of the forearm.

A 34-year-old male with lupus (not on any medications) and opioid use disorder, including injection drug use, presented to the emergency department with four days of swelling and pain in the right upper extremity (RUE) and chest wall after injecting heroin into the right elbow and missing a vein. He stated that the injection site became progressively more swollen and tender. He denied taking any medication for lupus. On hospital admission, the patient reported paresthesia and decreased sensation and range of motion of the right arm. The patient denied fever, chills, or shortness of breath. He was hypotensive (77/57 mmHg), tachycardic (108 bpm), and hypothermic (35.6°C). Laboratories revealed leukocytosis (21.4 x 10^3^/µL), polycythemia (21.4 g/dL), and thrombocytosis (781 x 10^3^/µL). On examination, the RUE showed noticeable edema, with pain during passive range of motion, decreased capillary refill, nonpalpable pulses, and inability to locate radial and ulnar signals. The right elbow showed notable areas of punctate ecchymosis and blistering, no crepitus, and tenderness up to the mid-upper arm. CT of the RUE revealed subcutaneous and intramuscular edema of the right forearm with a small focus of gas within the proximal radial aspect of the forearm (Figure 1).

These findings raised concerns about compartment syndrome with necrotizing soft tissue infection; thus, the decision was made to take the patient to the operating room (OR) for debridement and fasciotomy. He was empirically started on intravenous (IV) vancomycin, piperacillin/tazobactam, and clindamycin. 

In the OR, it was noted that the right dorsal forearm adjacent to the lateral epicondyle had soft tissue necrosis down to the subcutaneous tissue, with significant edema of the entire RUE. Compartment syndrome of RUE with myositis after decompressive fasciotomy was also noted. A second surgery was required the following day because of rapidly progressive infection that extended to the chest wall. The RUE fasciotomy was extended to the biceps. During surgery, the right chest wall was cut down, which ruled out necrotizing fasciitis despite severe edema in the area. After surgery, the patient became hypotensive and acidotic, with persistent, up-trending leukocytosis (41.2 x 10^3^/µL), and tachycardia, but remained afebrile. The patient was transferred to the surgical intensive care unit (ICU) for vasopressor support, and broad-spectrum antibiotics were continued. 

The patient continued to experience increased chest pain refractory to analgesics. Echocardiography revealed pericardial effusion with concern for cardiac tamponade. Upon prompt evaluation by the cardiology team, cardiac tamponade was ruled out. On day four of admission, intraoperative tissue cultures from the initial surgery revealed heavy growth of C. sordellii. Intraoperative cultures from the second surgery were finalized without growth. The antibiotics were modified to piperacillin/tazobactam as monotherapy. Later that day, the patient developed renal failure, requiring continuous renal replacement therapy. He also became progressively lethargic, requiring intubation, with refractory hypotension despite vasopressor therapy. His clinical status continued to deteriorate until the patient’s family opted for comfort measures and the patient died shortly thereafter. 

Case 2 

**Figure 2:**
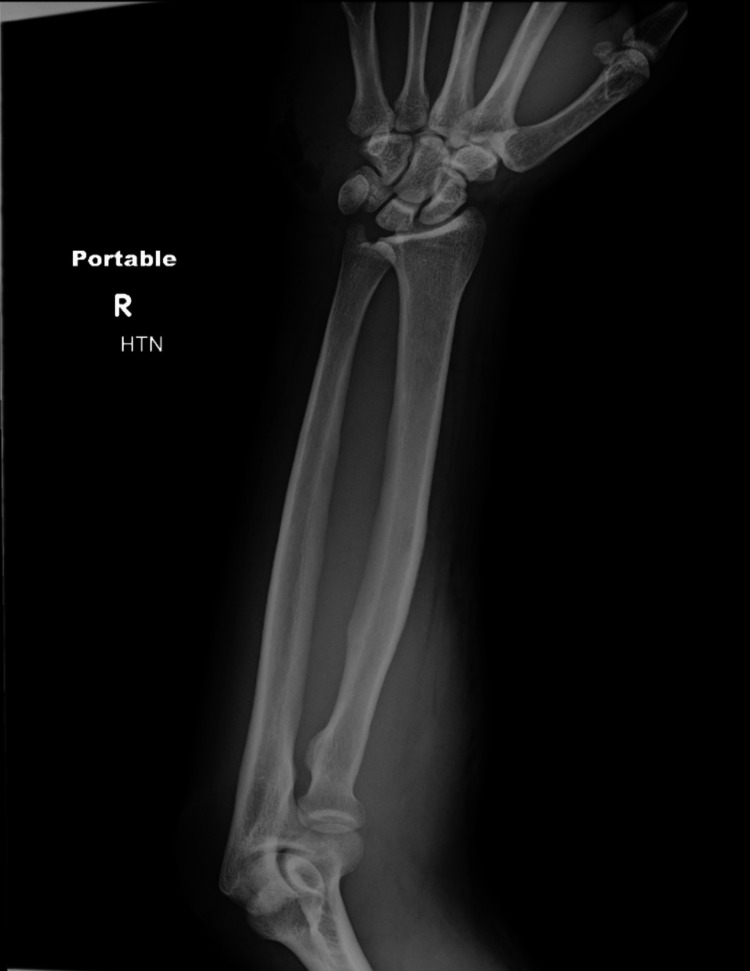
Frontal view of right forearm radiograph demonstrating soft tissue gas lucencies projecting over the medial hand.

A 41-year-old-male with methamphetamine use disorder, including injection drug use, presented to the emergency department with right hand and arm swelling and pain for three days. He mentioned incurring an injury to his hand five days prior while working in his car. On admission, he was hypotensive (76/32 mmHg), tachycardic (105 bpm), and afebrile, with an elevated lactate of 5.2 mmol/L, significant leukocytosis (49 x 10^3^/µL), and polycythemia (18.3 g/dL). The patient was alert and had experienced moderate acute distress at the time of admission. On examination, the right hand and forearm were diffusely edematous, weak, and tender. The posterior forearm was mottled purple and tense with patches of skin necrosis and scant serous drainage. The medial aspect of the right hand and distal forearm showed dusky discoloration, dry necrotic eschars with minimal blistering, and no surrounding erythema. All the extremities were cool to touch. The right radial pulse could not be palpated. Radiography of the right forearm revealed soft tissue gas lucencies projecting over the medial hand (Figure 2). 

Vasopressor support, intraosseous vancomycin, piperacillin/tazobactam, and clindamycin were administered immediately. Blood cultures were also obtained. The decision was made to take the patient emergently to the OR for cutting down, incision, and drainage of the right hand and forearm, given concern for necrotizing soft tissue infection with compartment syndrome. 

In the OR, an area of fluctuation was noted along the hypothenar eminence, which expressed purulent fluid. The fascia appeared intact, with the skin, soft tissue, and muscle excised from the area. The patient had significant tension in his hand and forearm, with concern for compartment syndrome; thus, orthopedic services were called to assist in the OR. They performed a right hand and forearm fasciotomy. Intraoperative tissue cultures were obtained. The patient was transferred to the surgical ICU for ongoing vasopressor support after surgery and broad-spectrum antibiotics were continued. 

The following day, the patient became more confused and restless. He developed an acute kidney injury (AKI), had persistent lactic acidosis, and had ongoing leukocytosis (35.2 x 10^3^/µL), with an ongoing vasopressor requirement to maintain hemodynamic stability. Blood and wound cultures did not demonstrate growth. Given the ongoing clinical instability, the patient was returned to the OR for further evaluation and debridement because of concerns of extensive progression of infection associated with possible new compartment syndrome of the upper arm proximal to the elbow. Volar superficial and deep fasciotomy was performed with additional excisional debridement of the right volar forearm, wrist, and hand. Significant edema was observed; however, no gross infection was detected along the fascial plane. The superficial volar fascia was incised and the underlying muscle appeared viable. Postoperatively, the patient was returned to the surgical ICU. 

On hospitalization day five, the patient was noted to have worsening clinical status with ongoing altered mentation requiring intubation. There was inability to palpate the right-hand pulse, and notable necrotic tissue in the wound bed. Additionally, the hypothenar muscle tissue culture from the initial surgery revealed C. sordellii growth. Blood cultures obtained on admission were finalized without growth. The patient was taken back to the operating room for further debridement, where he was found to have devitalized skin and dead muscle in the hypothenar area. This was sharply debrided slightly over the hypothenar eminence and along the dorsal aspect, eliminating the skin bridge between the previous fasciotomy incisions until healthy viable tissue was encountered. The patient remained critically ill after surgery, with ongoing hypotension requiring vasopressor support, and improved AKI after vancomycin discontinuation. Piperacillin/tazobactam and clindamycin were discontinued, ceftriaxone was initiated, and the patient completed 10 days of antibiotic treatment. Finally, a wound vacuum device was used to facilitate wound healing. 

After a 27-day hospitalization, the patient was discharged to a long-term acute care facility for ongoing recovery, with a planned outpatient follow-up with plastic surgery for wound management. 

Case 3 

**Figure 3:**
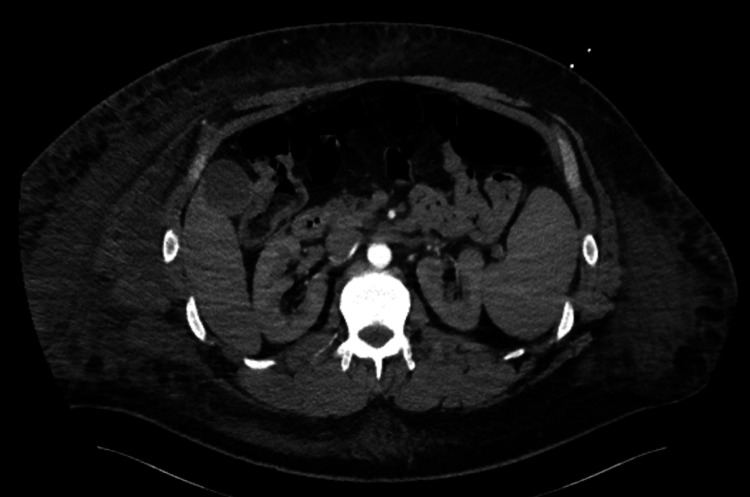
CT of the abdomen demonstrating subcutaneous edema with air pockets throughout the right lower quadrant abdominal wall.

A 42-year-old male with morbid obesity (body mass index, BMI: 34 kg/m^2^) and opioid use disorder, including injection drug use, presented to the emergency department of an outside hospital with complaints of abdominal pain that had persisted for approximately a week. The patient was intubated at the referral facility and started on IV vancomycin, piperacillin/tazobactam, and clindamycin. He developed shock that required vasopressor support. A CT scan of the abdomen from the referring facility showed a hiatal hernia without obvious evidence of gastric perforation as well as subcutaneous edema with air pockets throughout the right lower quadrant abdominal wall (Figure 3). 

Based on these findings, the patient was transferred to our hospital for high-level care. Upon examination, he was noted to have a large area of induration and erythema along the anterior right abdominal wall without palpable fluid collection, indicating a necrotizing soft tissue infection. He was afebrile in septic shock with profound leukocytosis (64.2 x 10^3^/µL) and polycythemia (18.4 g/dL). The patient underwent an emergency surgical intervention and debridement. 

In the OR, there were no drainable fluid pockets. However, the soft tissue underlying the skin was necrotic. The debridement was performed at the level of the anterior rectus sheath fascia. Following surgical intervention, the patient was transferred to the surgical ICU, where he continued to be profoundly hypotensive, requiring four vasopressors, stress-dose steroids, and methylene blue to maintain his blood pressure. The patient developed worsening renal and hypoxic respiratory failure, with hyperkalemia and acidosis. The patient was evaluated at bedside for debridement without further debridement. Ultimately, the patient’s family elected comfort care measures, and less than 24 h after presentation, the patient died. A few days later, an intraoperative abdominal wall tissue culture revealed *C. sordellii* growth.

Case 4 

A 47-year-old male with an opioid use disorder, including injection drug use, presented to an outside hospital with worsening right-arm swelling, pain, and erythema. He was started on IV piperacillin/tazobactam, vancomycin, and clindamycin and was transferred to our hospital for a higher level of care because of a concern for necrotizing fasciitis. On arrival, the patient stated that he had injected heroin into his right arm two-three days before symptom onset. He denied fever, but complained of pain in his right arm associated with chills. He was afebrile and normotensive on admission, with profound leukocytosis (39.5 x 10^3^/µL) and polycythemia (18.7 g/dL). On examination, the patient appeared to be confused and agitated. The RUE was tense and painful to touch, with areas of erythema and scattered lesions with a weeping yellow discharge throughout the right arm. The patient was immediately transferred to the operating room for surgical evaluation and intervention. 

**Figure 4:**
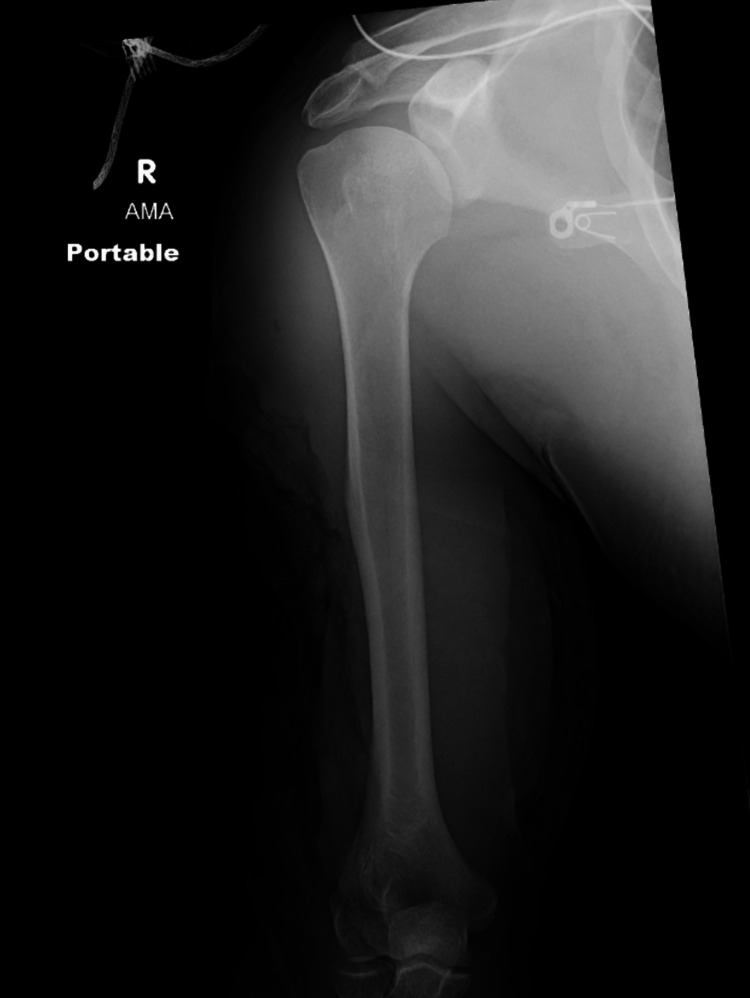
Radiography of the right arm demonstrating extensive soft tissue defects with overlapping soft tissue gas, equivocal for early osteomyelitis.

In the OR, fasciotomy of the right upper arm, forearm, and wrist and debridement of nonviable muscle tissue were performed. The patient also underwent a right dorsal hand fasciotomy. He was found to have myonecrosis of the right biceps muscle in the lateral head and right lateral triceps muscle, which were resected, suppurative right cephalic vein thrombophlebitis with abscess, and frank necrosis necessitating vein excision and compartment syndrome of the right forearm and upper arm without evidence of necrotizing fasciitis. The patient was transferred to the surgical ICU postoperatively, where he remained intubated and became hypotensive, requiring vasopressor support. Follow-up radiography of the right arm demonstrated extensive soft tissue defects with overlapping soft tissue gas, equivocal for early osteomyelitis (Figure 4). 

Intraoperative biceps muscle tissue cultures revealed growth of *C. sordellii* and *Bacillus cereus*. The patient remained on IV clindamycin, piperacillin/tazobactam, and vancomycin. 

**Figure 5:**
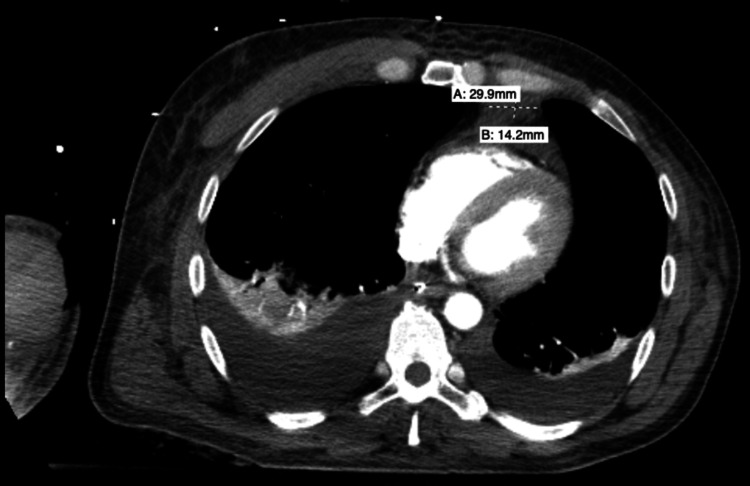
CT of the chest demonstrating a soft tissue nodular density in the anterior mediastinum measuring 3.0 cm x 1.4 cm, suggesting possible mediastinitis.

On admission day two, the patient continued to experience septic shock refractory to vasopressor support. A CT of the chest, abdomen, and pelvis was performed to evaluate other sources of infection, which demonstrated possible mediastinitis, with intramuscular fluid collection in the right external oblique muscle and asymmetric enlargement of the right pectoralis major and latissimus dorsi muscles, likely representing myositis (Figure 5). 

The patient returned to the OR for excisional debridement of the RUE, right posterior shoulder, right abdominal wall, and right buttock. He returned to the surgical ICU where he developed worsening renal failure with severe acidosis, requiring continuous renal replacement therapy. 

The patient continued to develop worsening shock, and a repeat CT scan was performed on day six of hospitalization, demonstrating improvement in the enlargement of the right trapezius, right latissimus dorsi, and right external oblique and bilateral gluteus maximus muscles with similar asymmetric enlargement of the right pectoralis major muscle without an obvious fluid collection. The patient returned to the OR for incision and drainage of the right pectoralis region and excisional debridement of the right anterior chest involving the skin, subcutaneous tissue, fascia, and pectoralis major. Right pectoralis major myosectomy was performed because of intraoperative findings of necrotizing myositis. The lateral head of the right biceps was non-viable. Necrotizing fasciitis was not observed. 

The patient remained critically ill with shock refractory to vasopressor support, stress-dose steroids, and evidence of multiorgan failure. Broad-spectrum antibiotics were continued. The following day, arterial blood gas analysis revealed metabolic acidosis with severely depressed neurological status. The palliative care team was consulted, and the goals of care discussions ensued with the family who opted for comfort care measures. The patient died shortly after.

## Discussion

Gas gangrene is a rapidly progressive, life-threatening infection of skeletal muscle caused by clostridia. Although NSTI are uncommon, they have been associated with high mortality and morbidity rates, with a global annual incidence of 0.3 to 15.5 cases per 100,000 people [5]. The usual incubation period between injury and the development of clostridial myonecrosis is two to three days but may be as short as 6 h [6]. Prompt recognition of this syndrome and appropriate surgical interventions are crucial for patient survival. We report four patients with NSTI, progressive myonecrosis, and fulminant shock caused by *C. sordellii*, whose main risk factors for infection were injection drug use. 

*Clostridium sordellii* is a well-known cause of fulminant infections in obstetric patients, but it is rarely reported as a cause of necrotizing soft tissue infections in other patient populations [7-8]. Individuals who inject drugs are at inherent risk of wound, skin, and soft tissue infections. Necrotizing fasciitis due to *C. sordellii* has been previously associated with the use of black tar heroin [2-3]. Similarly, a molecular epidemiological investigation in San Francisco, California, reported a clostridial myonecrosis cluster among injection drug users, where a common clonal strain was found in *C. sordellii* isolates [9]. In general, when clostridial necrotizing infection is suspected in injection drug users, the source of infection is usually abscesses or soiled skin at the injection site, contaminated injection devices, or illicit substances. 

*Clostridium sordellii* is a Gram-positive anerobe that is part of the normal human flora of the gastrointestinal tract and vagina. It produces up to seven identified exotoxins, two of which are lethal and hemorrhagic toxins, which are the major virulence factors [10]. Patients with reported *C. sordellii* infections are commonly afebrile and have refractory hypotension, hemoconcentration, and profound leukocytosis [10]. The hemoconcentration commonly associated with *C. sordellii* infections is attributed to capillary leakage from toxin-mediated changes to the endothelium [11]. Bullae formation, skin fluctuance and induration, crepitation, numbness, and necrosis are typically noted in the advanced stages of infection. 

In all four cases described, the patients presented with an advanced stage of infection, which allowed for clinical syndrome recognition and intervention. All our patients were afebrile and remained afebrile throughout most of their hospital courses. Nearly all patients, except one, developed septic shock. However, the patient progressed rapidly, requiring multiple vasopressors and refractory methods to maintain blood pressure. Additionally, all patients presented with hemoconcentration (18.3-21.4 g/dL) and profound leukocytosis (21.4-64.2 x 10^3^/µL). Leukocytosis remained profound throughout the hospital course and increased to a maximum of 73.1 x 10^3^/µL in one patient. This leukemoid reaction is a unique feature of clostridial infections and is highly correlated with increased mortality [11]. 

Diagnostic options are limited and the most important determinant of survival. In the early stages of infection, symptoms can easily be mistaken for cellulitis because they are non-specific. However, as the infection progresses, the clinical syndrome becomes more distinct, characterized by rapidly spreading edema, ecchymosis, bullae formation, and necrosis of the affected tissue [12]. Diagnostic imaging techniques such as radiography and CT may reveal the presence of gas in the affected tissues. However, their role in diagnosis is limited, and their findings tend to be nonspecific [12]. Therefore, caution should be exercised in causing further diagnostic delays owing to delays in imaging. Laboratory tests may show hemoconcentration and profound leukocytosis, both of which are highly suggestive of clostridial infection [11]. Prompt surgical debridement of the infected necrotic tissue is critical, as antibiotic therapy alone is typically ineffective owing to the rapid spread and toxin-mediated pathogenesis of clostridial myonecrosis. Owing to the known limitations in the diagnosis of this rapidly fatal infection, it is important to consider all available resources to establish the diagnosis and promptly initiate appropriate treatment. 

Treatment of *C. sordellii* necrotizing soft tissue infections requires emergent surgical intervention. Antibiotic treatment alone will not suffice, and should never replace aggressive surgical debridement [13-14]. Most *C. sordellii* strains are susceptible to β-lactam antibiotics. Adjunctive therapy with clindamycin for the suppression of toxin production, hyperbaric oxygen, or IV immunoglobulin remains a question [15]. Although all our patients received empiric clindamycin therapy, this was done prior to being aware of the causative bacterial pathogen, and in the event that the necrotizing infection was due to *Streptococcus pyogenes*. 

Clinicians should consider NSTI as part of the differential diagnosis during the evaluation of skin and soft tissue infections in injection drug users. In our clinical experience, routine complete blood cell count analysis may indicate a diagnosis, particularly marked leukocytosis, in addition to a high hemoglobin count in an afebrile patient. Notably, all four patients also developed fulminant septic shock refractory to vasopressor therapy. Early recognition of symptoms and timely aggressive treatment may improve survival in these patients. Therefore, patients who inject drugs should be routinely informed of the risks of NSTI and warning signs to expedite medical attention.

## Conclusions

This case series illustrates the rapid progression of *C. sordellii* NSTI in people who inject drugs and the need for prompt surgical debridement and antibiotic therapy at the first signs of infection. The association between *C. sordellii* and injection drug use suggests that either contaminated illicit substances or subsequent injection techniques may serve as reservoirs for this pathogen. This highlights that *C. sordellii* NSTI remains an important cause of morbidity and mortality in patients who inject drugs, underscoring the need for increased awareness. Further research is needed to understand the transmission dynamics of *C. sordellii* in this vulnerable patient population and to explore prevention strategies along with methods of early detection. Additionally, awareness campaigns aimed at educating patients and healthcare providers on the risks and importance of early intervention may have a valuable impact on the mortality associated with this infection. Early recognition, together with aggressive surgical and medical approaches, is imperative for survival.
